# Characterization of host factors associated with the internal ribosomal entry sites of foot-and-mouth disease and classical swine fever viruses

**DOI:** 10.1038/s41598-022-10437-z

**Published:** 2022-04-25

**Authors:** Yutaro Ide, Bouchra Kitab, Nobumasa Ito, Riai Okamoto, Yui Tamura, Takafumi Matsui, Yoshihiro Sakoda, Kyoko Tsukiyama-Kohara

**Affiliations:** 1 Transboundary Animal Disease Center, Joint Faculty of Veterinary Medicine, Kagoshima University, Kagoshima, Japan; 2 Laboratory of Animal Hygiene, Joint Faculty of Veterinary Medicine, Kagoshima University, 1-21-24, Korimoto, Kagoshima, 890-0065 Japan; 3 Department of Disease Control, Faculty of Veterinary Medicine, Hokkaido University, Hokkaido, 060-0818 Japan

**Keywords:** Microbiology, Molecular biology

## Abstract

Foot-and-mouth disease virus (FMDV) and classical swine fever virus (CSFV) possess positive-sense single-stranded RNA genomes and an internal ribosomal entry site (IRES) element within their 5′-untranslated regions. To investigate the common host factors associated with these IRESs, we established cell lines expressing a bicistronic luciferase reporter plasmid containing an FMDV-IRES or CSFV-IRES element between the *Renilla* and firefly luciferase genes. First, we treated FMDV-IRES cells with the French maritime pine extract, Pycnogenol (PYC), and examined its suppressive effect on FMDV-IRES activity, as PYC has been reported to have antiviral properties. Next, we performed microarray analysis to identify the host factors that modified their expression upon treatment with PYC, and confirmed their function using specific siRNAs. We found that polycystic kidney disease 1-like 3 (PKD1L3) and ubiquitin-specific peptidase 31 (USP31) were associated with FMDV-IRES activity. Moreover, silencing of these factors significantly suppressed CSFV-IRES activity. Thus, PKD1L3 and USP31 are host factors associated with the functions of FMDV- and CSFV-IRES elements.

## Introduction

Foot-and-mouth disease (FMD) and classical swine fever (CSF) are highly contagious viral diseases that affect cloven-hoofed animals^1^ and swine^2^, respectively. The causative pathogens of these diseases, the FMD virus (FMDV; genus *Aphthovirus*, family Picornaviridae) and CSF virus (CSFV; genus *Pestivirus*, family Flaviviridae), possess positive-sense, single-stranded RNA genomes. In both genomes, translation of the virus-encoded polyprotein is directed by an internal ribosomal entry site (IRES) within a relatively long 5ʹ-untranslated region (5ʹ-UTR). FMDV-IRES is classified as a type II IRES, which is also observed in the 5ʹ-UTR of cardioviruses (e.g., encephalomyocarditis virus)^3^. CSFV-IRES is classified as a hepatitis C virus (HCV)-like IRES^4,5^. In an HCV-like IRES, domains II-IV are required for IRES activity, and HCV-like IRESs are shorter and more compact than type I and II picornavirus IRESes^6^. Common host factors (other than the canonical translation initiation factors) essential for the activity of these IRESs have not been fully characterized to date^7,8^ except for the proteomic analysis of interacting proteins^9,10^.

The French maritime pine extract Pycnogenol (PYC; a registered trademark of Horphag Research, Geneva, Switzerland) was produced from the outer bark of a maritime pine tree (*Pinus pinaster* ssp. *atlantica*) and is a promising therapeutic agent with potential applications in human health (American Botanical Council, 2010; http://herbalgram.org/resources/herbclip/herbclip-news/2010/rye/). PYC inhibits encephalomyocarditis virus replication in the mouse heart by suppressing the expression of proinflammatory cytokines^11^ and inhibits the binding of human immunodeficiency virus type-1 to cells^12^. Moreover, PYC exhibited an inhibitory effect on HCV replication in vitro and in vivo^13^.

In this study, we aimed to identify the host factors associated with the activity of both FMDV-IRES and CSFV-IRES by identifying the genes that were differentially expressed after PYC treatment. Subsequently, we performed comprehensive analyses using short interfering RNA (siRNAs).

## Materials and methods

### Cell culture, virus, and plasmids

The human kidney cell line (HEK293) used in this study was obtained and cultured as previously described^14,15^. The swine kidney line L (SK-L) cells were propagated in Eagle’s Minimum Essential Medium (Nissui Pharmaceutical, Tokyo, Japan) supplemented with 0.3 mg/mL l-glutamine (Nacalai Tesque, Kyoto, Japan), 100 U/mg penicillin G (Meiji Seika Pharma, Tokyo, Japan), 8 mg/mL gentamycin (TAKATA Pharmaceutical, Saitama, Japan), sodium bicarbonate (Nacalai Tesque), 0.1 mg/mL streptomycin (Meiji Seika Pharma), 0.295% tryptose phosphate broth (Becton Dickinson and Company, Franklin Lakes, NJ, USA), 10 mM *N,N*-bis-(2-hydroxyethyl)-2-aminoethanesulfonic acid (BSE; MilliporeSigma, St. Louis, MO, USA), and 10% horse serum (Thermo Fisher Scientific, Waltham, MA, USA).

The vCSFV GPE^-^/HiBiT recombinant classical swine fever virus encoding the HiBit luciferase gene^16^ was derived from the recombinant full-length cDNA of the CSFV GPE^-^strain^17^. SK-L cells were infected with tenfold serially diluted vCSFV GPE-HiBiT in 96-well plates and incubated at 37 °C for 3 days. Virus growth was analyzed using luciferase activity as an indicator. Viral titers were calculated and expressed as the tissue culture infectious dose (TCID_50_) per mL. The luciferase assay was performed according to a previously described protocol^18^. The luciferase activity of the culture supernatants was measured using a Nano-Glo HiBiT lytic detection system (Promega, Madison, WI, USA) according to the manufacturer’s protocol. Twenty µL of culture medium was mixed with an equal volume of Nano-Glo HiBiT lytic buffer. Luciferase activity was measured in a 96-well LumiNunc™ plate (Thermo Fisher Scientific) using the microtiter plate reader POWERSCAN 4 (DS Pharma Biomedical, Osaka, Japan). The average number of mock-infected 96-well plates plus five times the standard deviation of this population (i.e., luciferase activity = 70) was set as the cutoff value.

The pRF vector containing an FMDV-IRES (serotype C; 5ʹ-UTR sequence: nucleotides (nt) 569–1038 in FMDV serotype C, AF274010.1)^19^ was kindly donated by Dr. Hirasawa of the Memorial University of Newfoundland, and those containing a CSFV-IRES^20^ were gifts from Professor Graham J. Belsham of the University of Copenhagen. The pCAGGS-Neo vector was constructed using the pCAG Neo (Fujifilm Wako, Tokyo, Japan) and pCAGGS vectors (Cat. No. RDB08938; Riken Bank, Ibaraki, Japan). The CSFV-IRES cDNA (nt. 124–401) was excised from a reporter plasmid^20^ using the *Eco*RI and *Nco*I restriction sites, and the excised DNA was inserted between the *Renilla* and firefly luciferase genes. Reporter genes were cut using the restriction endonucleases *Eco*RV (Toyobo, Osaka, Japan) and *Bam*HI (New England Biolabs, Ipswich, MA, USA). A pCAGGS-Neo/CSFV-IRES vector was generated by inserting a reporter gene into pCAGGS-Neo, which was subsequently treated with *Eco*RV (Toyobo), *Bam*HI (New England Biolabs), and rAPid alkaline phosphatase (Roche, Basel, Switzerland) using a ligation mixture (Mighty Mix, Takara, Shiga, Japan).

Cells expressing pCAGGS-Neo-CSFV-IRES (clones pCI5 and pCI5-1) and pCAGGS-Neo-FMDV-IRES (clones B5 and B10) were established as described previously^15^.

DNA sequencing was performed by FASMAC Co. (Kanagawa, Japan), and DNA sequence characterization was performed using the GENETYX-Mac software (GENETYX Co., Tokyo, Japan) and GENBANK.

Cell viability was evaluated using WST assays (Dojindo, Kumamoto, Japan) by determining the optical density at 450 nm (OD_450_) according to the manufacturer’s instructions. Luciferase assays were performed using a dual-luciferase reporter assay system (Promega, Madison, WI, USA). Luminescence was measured using a GloMax 96 Microplate Luminometer (Promega) for 10 s, as described previously^14^.

### RNA isolation and microarray analysis

Total RNA was extracted from PYC-treated (10 μg/mL, 72 h) and untreated B10 cells using ISOGEN (Nippon Gene Co. Tokyo, Japan) from cells growing in the linear phase of PYC treatment. RNA quality was measured using an Agilent 2100 bioanalyzer and showed an RNA integrity number (RIN) of 9.8 (7.0 < RIN ≤ 10 is suitable for analysis). Microarray analysis was performed by Hokkaido System Science Co., Ltd. (Sapporo, Japan) using the SurePrint G3 Human 8 × 60 K ver 3.0 slides (Agilent Technologies Co., Santa Clara, CA, USA). RNA samples were labeled with Cy3 or Cy5, hybridized on slides using a gene expression hybridization kit (Agilent Technologies Co.), washed with gene expression wash buffer (Agilent Technologies Co.), and scanned using a microarray scanner (G2505C, Agilent Technologies Co.). Raw images were processed using the Agilent Feature Extraction software (12.0.3.1).

### Quantitative real time PCR (qRT-PCR)

The amounts of *PKD1L3* and *USP31* mRNA in cells were quantified using the SYBR Green real-time PCR master mix (Thermo Fisher, Waltham, MA, USA) and the primers pKD1L3-544S: 5ʹ-CATCTTCCAACCACATGTCACTATCC-3ʹ, pKD1L3-903AS: 5ʹ-CTGTAGTTTGTTAAGAGCTTGCAAACC-3ʹ; USP31-700S: 5ʹ- TGTGGCTTTTGGACCGAGTTGC-3ʹ, and USP31-900AS: 5ʹ- TGCAGTGAGAACATTTGCCTGC-3ʹ. The data was evaluated using the 2^ΔΔ−Ct^ method.

### siRNA transfection

The siRNAs targeting host factors (summarized in Table 2) were designed using the BLOCK-iT RNAi Designer (Thermo Fisher Scientific, Waltham, MA, USA). For the control siRNA, an ON-target plus siRNA control (Horizon/Dharmacon, Lafayette, CO, USA) was used. Then, siRNA (5 nM) reverse transfection was performed using the Lipofectamine RNAiMAX reagent (Invitrogen) according to the manufacturer’s specifications. The effect of siRNA was evaluated by immunoblot analysis as described previously^14^ using anti-polycystic kidney disease 1-like 3 (PKD1L3) (OSP00014W, Invitrogen) and anti-ubiquitin-specific peptidase 31 (USP31) (Santa Cruz Biotechnology Co.) antibodies. Original blots presented in the supplementary original gel image_Fig. 5 which shows fuller-length of both sides and bottom, but top stacking part gel was removed.

### Statistical analysis

All data are presented as mean ± standard deviation (S.D.) from three independent experiments, and figures were generated using GraphPad Prism (version 9) software. Statistical analysis was performed using Student’s *t-*test to evaluate significant differences. Statistical significance was set at *P* < 0.05.

### Ethics declarations

This study was performed in accordance with institutional committee protocols of Kagoshima University.

## Results

### Effect of PYC on FMDV-IRES activity

**Figure 1:**
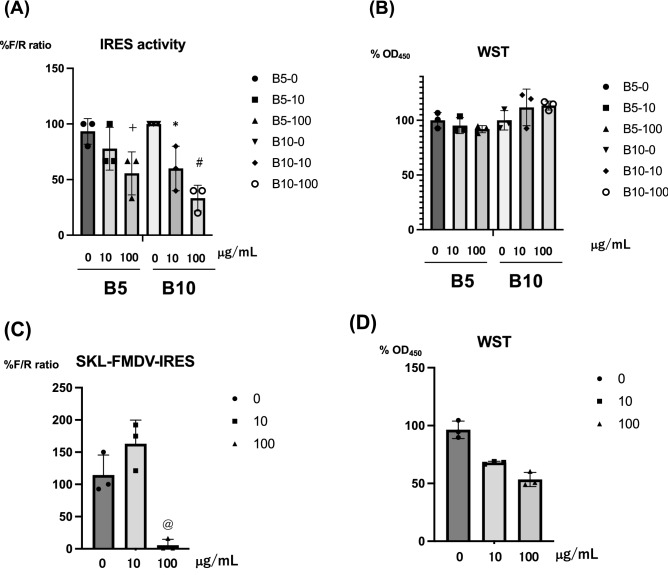
Treatment of FMDV-IRES expressing cells with PYC. **(A)** FMDV-IRES-expressing B5 and B10 cells were treated with PYC at final concentrations of 0, 10, and 100 μg/mL. After 72 h of incubation, firefly and *Renilla* luciferase activities were evaluated, and IRES activity was calculated as the ratio of firefly luciferase activity to *Renilla* luciferase activity, plotted against the value for the untreated sample. + *P* = 0.022, *P = 0.037, #*P* = 0.0049 (**B**) The ratio (%) of the WST value (OD_450_) versus the untreated sample is shown. FMDV-IRES expressing SK-L cells were treated with PYC and the luciferase activity (**C**) and WST values (**D**) were evaluated. Bars and vertical bars indicate the mean values of the triplicate samples and S.D, respectively. *P* values indicate statistical significance. ^@^*P* = 0.017. *FMDV* foot-and-mouth disease virus, *IRES* internal ribosomal entry site, *PYC* pycnogenol.

To identify the common host factors associated with FMDV-IRES and CSFV-IRES, we characterized the effect of PYC on FMDV-IRES activity, as PYC is a natural product that has been reported to show antiviral effects against a few viruses^11,13,21^. B5 and B10 cells that express bicistronic dual-luciferase mRNAs containing FMDV IRES were treated with PYC^15^ for 72 h. As shown in Fig. 1A, FMDV-IRES activity was significantly suppressed in a dose-dependent manner, and no significant cytotoxicity was observed (Fig. 1B). We also examined the effect of PYC on FMDV-IRES in swine cells (SK-L) after transfection with the pRF plasmid^15^ and observed a suppressive effect on IRES activity (Fig. 1C and D).

To identify the host factors affected by PYC treatment, we treated B10 cells with PYC and analyzed differential gene expression using microarray analysis. We identified 115 downregulated genes and 218 upregulated genes with a more than two-fold change after PYC treatment (Supplementary Table 1). We selected the top 10 genes (Table 1, No. 1–10, except for pseudogene [No. 3]), which were downregulated after treatment with PYC, to design siRNAs (Table 2) and characterize their functions in IRES activity.

**Table 1:** Result of microarray analysis (top 10 downregulated genes by PYC treatment).

No.	GeneName	SystematicName	Accessions	Chr_coord	Description	FoldChange*	PValueLogRatio	gProcessedSignal	rProcessedSignal
1	lnc-IFI44-2	lnc-IFI44-2:4	linc|lnc-IFI44-2:4|linc|lnc-IFI44-2:5|linc|lnc-IFI44-2:3|linc|TCONS_00002078	hs|chr1:79869806-79871942	linc|LNCipedia lincRNA (lnc-IFI44-2), lincRNA [lnc-IFI44-2:4]	1.50E+01	9.72E−15	8.31E+01	5.54E+00
2	**PKD1L3**	NM_181536	ref|NM_181536|ens|ENST00000620267|linc|lnc-ZNF821-2:1|linc|lnc-ZNF821-2:2	hs|chr16:71963542-71963483	ref|Homo sapiens polycystic kidney disease 1-like 3 (PKD1L3), mRNA [NM_181536]	1.16E+01	8.55E−12	6.28E+01	5.43E+00
3	LOC100129935	NR_026870	ref|NR_026870|ens|ENST00000597791|ens|ENST00000597077|ens|ENST00000594263	hs|chr19:40131243-40131302	ref|Homo sapiens lectin, galactoside-binding, soluble, 14 pseudogene (LOC100129935), non-coding RNA [NR_026870]	6.81E+00	9.66E−04	2.23E+01	3.27E+00
4	ENST00000456414	ENST456414	ens|ENST00000456414|linc|lnc-CD58-1:1|gb|AL832882|gb|AK126833	hs|chr1:117035719-117035660	gb|Homo sapiens mRNA; cDNA DKFZp667K053 (from clone DKFZp667K053). [AL832882]	5.03E+00	7.27E−04	2.61E+01	5.19E+00
5	lnc-SGTB-2	lnc-SGTB-2:1	linc|lnc-SGTB-2:1|tc|THC2736569	hs|chr5:65440279-65440220	tc|K1C14_MOUSE (Q61781) Keratin, type I cytoskeletal 14 (Cytokeratin-14) (CK-14) (Keratin-14) (K14), partial (5%) [THC2736569]	4.69E+00	2.13E−05	3.60E+01	7.67E+00
6	ENST00000455576	ENST455576	ens|ENST00000455576|linc|lnc-RARB-1:1|gb|DA240288	hs|chr3:25215706-25215765	gb|DA240288 BRAWH3 Homo sapiens cDNA clone BRAWH3037815 5', mRNA sequence [DA240288]	4.25E+00	3.06E−03	2.11E+01	4.97E+00
7	**SLC24A4**	NM_153646	ref|NM_153646|ref|NM_153647|ref|NM_153648|ens|ENST00000531433	hs|chr14:92962507-92962566	ref|Homo sapiens solute carrier family 24 (sodium/potassium/calcium exchanger), member 4 (SLC24A4), transcript variant 1, mRNA [NM_153646]	4.19E+00	9.53E−04	2.57E+01	6.13E+00
8	**USP31**	NM_020718	ref|NM_020718|ens|ENST00000219689|linc|lnc-COG7-2:1|ref|XM_005255450	hs|chr16:23074153-23074094	ref|Homo sapiens ubiquitin specific peptidase 31 (USP31), mRNA [NM_020718]	4.10E+00	1.51E−03	2.70E+01	6.60E+00
9	ENST00000554254	ENST554254	ens|ENST00000554254|linc|lnc-TMEM30B-5:1|tc|THC2667238|tc|THC2685569	hs|chr14:62217662-62217603	ens|HIF1A antisense RNA 2 [Source:HGNC Symbol;Acc:HGNC:43015] [ENST00000554254]	4.09E+00	2.26E−04	3.01E+01	7.35E+00
10	**HGF**	NM_001010934	ref|NM_001010934|ens|ENST00000423064|gb|AB208900|gb|U46010	hs|chr7:81380346-81380287	ref|Homo sapiens hepatocyte growth factor (hepapoietin A; scatter factor) (HGF), transcript variant 5, mRNA [NM_001010934]	4.04E+00	7.73E−10	8.83E+01	2.19E+01

*Top 10 downregulated genes (fold decrease) by PYC treatment were shown. Gene name, systematic name, accession name etc has been indicated.

**Table 2:** List of siRNAs targeting host factors.

Name of host factor	siRNA sequence
1. lnc-IFI44-2	5’-CCAAUGCUGUGAGAGUUGUACAUGU-3’
2. PKD1L3	5′- CAGUUCAUGGUUUGCAAGCUCUUAA-3′,
4. ENST456414	5’-GAGGAGGGAAGAGAAUGAATCUUAU-3’
5. lnc-SGBT-2	5’-CAGUGCCCAUGUUUCUUGUGUUUAA-3’
6. ENST455576	5’-UGCAGUGCAUUUGCCUCCCUCACUU-3’
7. SLC24A4	5’-GACGGUAGCUAUGAUGACCCUUCCG-3’
8. USP31	5′- CAGCACAGCCGCGACUUCAAGACUA-3′
9. ENST554254	5’-ACAGGUCAAGUGAAGUUCUUCUGCU-3’
10. HGF	5’-GGGACCCUGGTGUUUCACAAGCAAU-3’

### Characterization of the role of host factors in FMDV-IRES-activity

**Figure 2:**
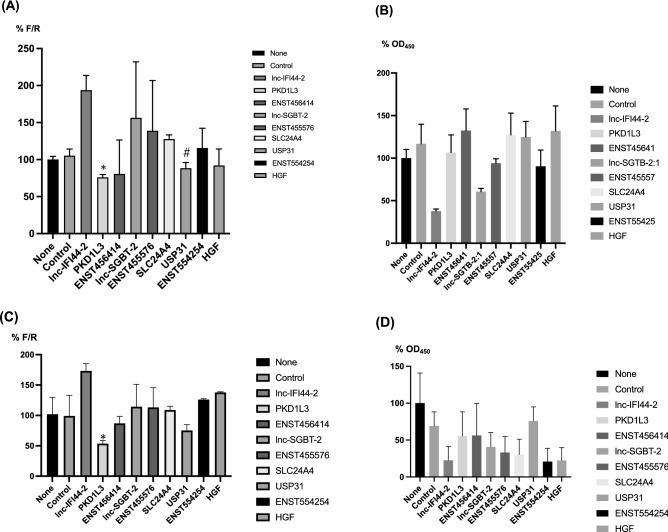
Transfection of siRNA into FMDV-IRES expressing cells. siRNA targeting nine upregulated genes (Table 1) was reverse transfected into FMDV-IRES-expressing cells B10 (**A**) and B5 (**C**). The IRES activity was evaluated as shown in Fig. 1. Statistical analysis was performed using the Student’s *t*-test to compare the control siRNA-treated cells and cells transfected with siRNA targeting host factors. *P = 0.011, ^#^*P* = 0.021 **(B,D)** The ratio (%) of the WST value (OD_450_) versus untreated sample in B10 (**B**) and B5 (**D**) cells. *P = 0.021. Bars and vertical bars indicate the mean values of triplicate samples and SD, respectively.

To further investigate the role of the candidate host factors (downregulated genes identified as described above), we targeted each gene with a specific siRNA (Table 2) in cell lines expressing bicistronic reporter mRNA containing FMDV-IRES^14^ (Fig. 2). Silencing *PKD1L3* and *USP31* significantly suppressed FMDV-IRES activity (Fig. 2A) without inducing cytotoxicity (Fig. 2B). We also confirmed the effect of PYC on *PKD1L3* and *USP31* using qRT-PCR and observed downregulation of gene expression (Supplementary Fig. S1).

### Establishment of CSFV-IRES-expressing cell lines

**Figure 3:**
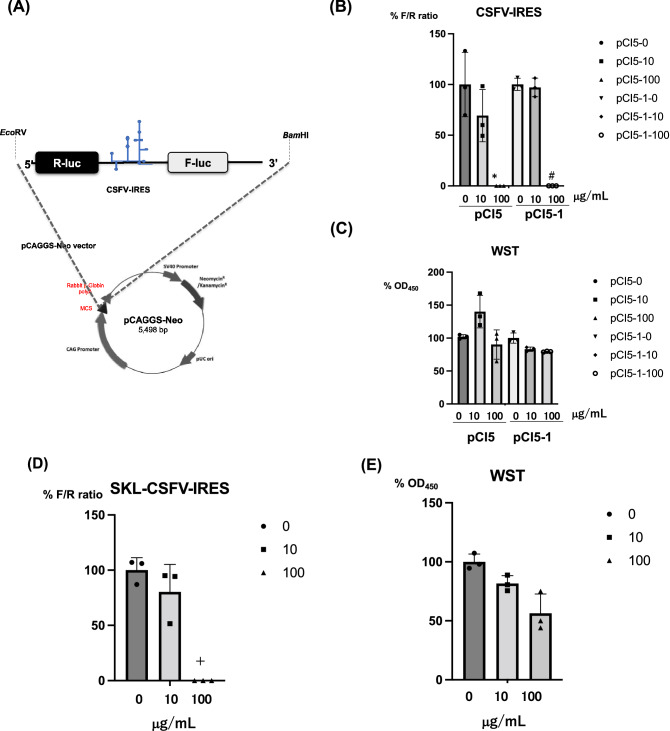
Structure of the bicistronic luciferase reporter construct and response to PYC. **(A)** A bicistronic reporter construct was designed to contain the CSFV-IRES element located between the *Renilla* and firefly luciferase genes. The bicistronic reporter gene was excised using the restriction enzymes *Eco*RV and *Bam*HI and ligated into the pCAGGS-Neo/MCS vector digested with *Eco*RV and *Bam*HI. **(B)** CSFV-IRES-expressing cell lines (pCI5 and pCI5-1) were treated with PYC at final concentrations of 0, 10, or 100 μg/mL. After 72 h of incubation, the firefly and *Renilla* luciferase activities were evaluated, and IRES activity was calculated as the ratio of firefly to *Renilla* luciferase activity plotted against the value for the untreated sample. *P = 0.016, #*P* = 0.00061 **(C)**The ratio (%) of the WST value (OD_450_) vs. the untreated sample is shown. CSFV-IRES expressing SK-L cells were treated with PYC and the luciferase activity (**D**) and WST values (**E**) were evaluated. ^+^*P* = 0.0021 The mean value for triplicate samples is indicated, and the vertical bars show SD. CSFV, classical swine fever virus.

**Figure 4:**
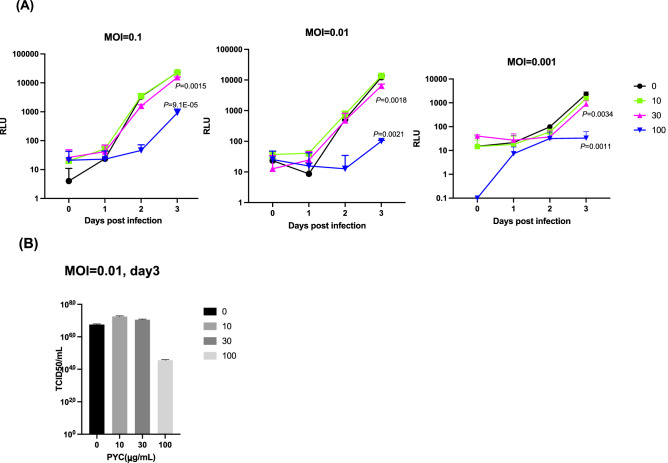
Effect of PYC to CSFV infection. (**A**) SK-L cells in triplicate wells (6 well plates) were infected with vCSFV GPE^−^/HiBiT at a multiplicity of infection (MOI) of 0.1, 0.01, and 0.01, treated with PYC at 10, 30, 100 mg/mL and analyzed daily. Significant differences relative to PYC 0 mg/mL treatment at day3 are indicated using *P*-values. (**B**) SK-L cells were infected with diluted vCSFV GPE^−^/HiBiT at a MOI = 0.01 in 96-well plates and incubated at 37 °C for 3 days. Virus growth was evaluated using the luciferase assay as described in the “Materials and methods”. Virus titers were calculated and expressed as TCID_50_ per mL.

FMDV-IRES is classified as a type II picornavirus IRES, whereas CSFV-IRES is classified as an HCV-like IRES (Supplementary Fig. S2)^6^. To evaluate CSFV-IRES activity, we established the cell lines pCI5 and pCI5-1 that incorporate a bicistronic reporter plasmid containing CSFV-IRES^20^ using the pCAGGS vector^14^ (Fig. 3A). We further treated CSFV-IRES-expressing cells with PYC to identify host factors that may be involved. Treatment with PYC suppressed CSFV-IRES activity in a dose-dependent manner (Fig. 3B), without inducing significant cytotoxicity (Fig. 3C). We also confirmed the effect of PYC on CSFV-IRES activity in swine cells and observed a suppressive effect (Fig. 3D,E). The effect of PYC on CSFV infection was examined using vCSFV GPE^-^/HiBiT, and a suppressive effect was observed in the results of the luciferase assay and TCID_50_, as described in the Materials and Methods section (Fig. 4). The propagation of CSFV was suppressed by PYC in a dose-dependent manner (Fig. 4A,B).

### Role of host factors in IRES-mediated translation of CSFV

**Figure 5:**
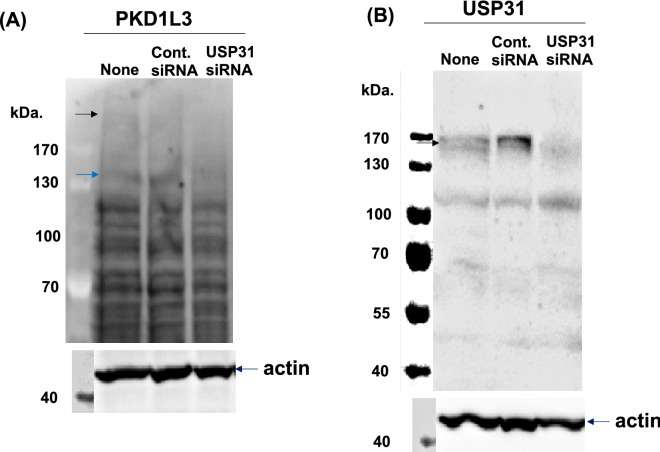
Silencing of target protein expression by siRNA. The effects of siRNA targeting against *PKD1L3* and *USP31* were examined using immunoblot analysis. Expression of the **(A)** PKD1L3 protein (~ 234 KDa, 150 KDa) and **(B)** USP31protein (~ 147 KDa) was evaluated (indicated by arrows). An anti-actin antibody was used as a loading control. Protein molecular weight markers (ThermoFisher Co.) are also shown. Original gel image is show in Supplementary Information.

The effect of siRNA targeting *PKD1L3* and *USP31* was examined by immunoblot analysis (Fig. 5). Treatment with these siRNAs suppressed the expression of target proteins (the full-length mature form of PKD1L3 is ~ 234 kDa^22^, and that of USP31 is ~ 120–140 kDa^23^).

**Figure 6:**
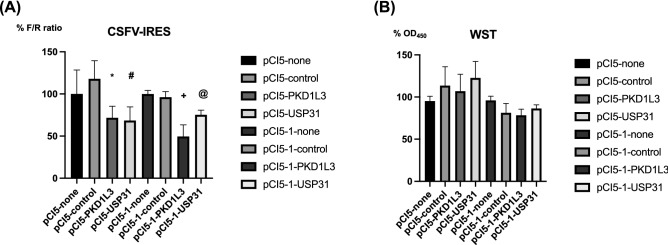
Effects of siRNA silencing of *PKD1L3* and *USP31* on CSFV-IRES activity. siRNAs targeting *PKD1L3* and *USP31* were reverse transfected into pCI5 and pCI5-1 cells using Lipofectamine RNAiMAX and incubated for 72 h. Firefly (CSFV-IRES activity) and *Renilla* (cap-dependent translation) luciferase activities were analyzed. **(A)** To evaluate IRES-mediated translational activity, the ratios of IRES-mediated translation vs. cap-dependent translation and vs. translation in untreated cells were calculated. Bars and vertical bars indicate the mean values of the triplicate samples and S.D, respectively. The Student’s *t*-test was performed to calculate *P*-values to compare control siRNA-treated cells and cells transfected with *PKD1L3* and *USP*31 siRNA. *P = 0.022, ^#^*P* = 0.012, ^+^*P* = 0.013, ^@^*P* = 0.01 (**B**) Cell viability was measured using WST assays by determining the OD_450_ and the ratios to the values for untreated cells. The experiments were performed in triplicate, and error bars indicate S.D.

To investigate the host factors associated with the activity of the IRES, we targeted *PKD1L3* and *USP31* with siRNA in two CSFV-IRES-expressing cell lines (Fig. 6) because these host factors are also involved in FMDV-IRES activity (Fig. 2). Silencing *PKD1L3* and *USP3*1 significantly suppressed CSFV-IRES activity in both cell lines (Fig. 6A) without inducing cytotoxicity (Fig. 6B).

## Discussion

In this study, we evaluated the suppressive effects of PYC on the activities of FMDV-IRES and CSFV-IRES. The results indicate that PYC may exert antiviral effects against FMDV and CSFV. We previously analyzed the antiviral effect of PYC against HCV infection^13^. PYC suppressed HCV replication in subgenomic replicon cells and infection in HCV-JFH-1^24^ infected cells, showed synergistic effects against interferon-α and ribavirin, and was effective against viruses resistant to direct-acting antiviral agents in vitro and in humanized chimeric mice^25^. PYC is a natural product; therefore, future studies should address the effects of PYC in FMDV infections.

After ascertaining the suppressive effects of PYC on FMDV-IRES and CSFV-IRES activities, we found that PKD1L3 and USP31 may be common host factors associated with the functions of FMDV-IRES and CSFV-IRES. To the best of our knowledge, this is the first report linking PKD1L3 and USP31 with IRES functions.

PKD1L3 was first identified as a candidate regulator of sour taste^26,27^. PKD1L3 cleaves the N-terminal G-protein-coupled receptor proteolytic site^28^ and reportedly forms a complex with PKD2-L1 and regulates the influx of Ca^2+^ to produce a Ca^2+^ spike during sensory responses^29^. USP31 is a deubiquitinating enzyme that activates nuclear factor-kappa B^30^. USP31 variants are strongly associated with adult-onset deafness in Border Collies^31^. The role of PKD1L3 and USP31 in IRES activity should be addressed in future studies.

In summary, IRES-mediated translational activity of FMDV and CSFV may be a suitable target for the development of a wide range of antiviral drugs because of the relatively high sequence conservation of IRES in each virus. Compounds that suppress PKD1L3 and USP31 can be developed as new anti-FMDV and CSFV drugs, and the downregulation of *PKD1L3* and *USP31* expression may contribute to the establishment of FMDV-and CSFV-resistant animal breeds.

## Supplementary Information

Supplementary Information.

Supplementary Table 1.

Supplementary Figures.

## Abbreviations

FMDV: Foot-and-mouth disease virus
CSFV: Classical swine fever virus
IRES: Internal ribosomal entry site
5UTR: 5′ Untranslated region
siRNA: Short interfering RNA
PYC: Pycnogenol
PKD1L3: Polycystic kidney disease 1-like 3
USP31: Ubiquitin-specific peptidase 31
