# Supplementary figures and images for: Characterization of host factors associated with the internal ribosomal entry sites of foot-and-mouth disease and classical swine fever viruses
Source: Sci Rep. 2022 Apr 25;12:6709. doi: 10.1038/s41598-022-10437-z (PMC9039067; doi:10.1038/s41598-022-10437-z)

USP31 (←)

non Cont. USP31 si USP31 si (pCI5 cells)

KDa.

170 —

130 —

100 —

70 —

55 —

40 —

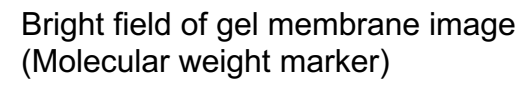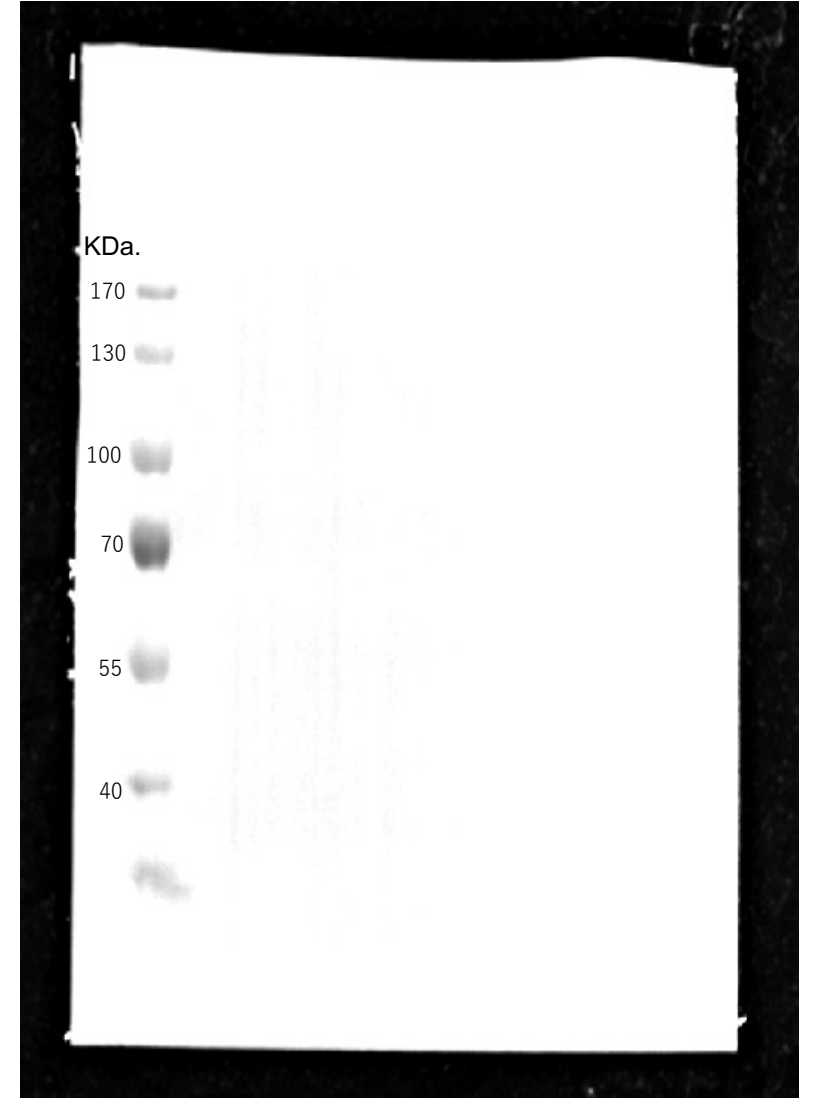

Supplement: Supplementary file 1 — Supplementary Information. [file 41598_2022_10437_MOESM1_ESM.pdf]
