# Supplementary material for: Characterization of host factors associated with the internal ribosomal entry sites of foot-and-mouth disease and classical swine fever viruses
Source: Sci Rep. 2022 Apr 25;12:6709. doi: 10.1038/s41598-022-10437-z (PMC9039067; doi:10.1038/s41598-022-10437-z)
Supplement: Supplementary file 3 — Supplementary Figures. [file 41598_2022_10437_MOESM3_ESM.pdf]

Supplementary Information

Characterization of host factors associated with the internal ribosomal entry sites of foot-and-mouth disease and classical swine fever viruses

Yutaro Ide[1], Bouchra Kitab[1], Nobumasa Ito[1], Riai Okamoto[1], Yui Tamura[1], Takafumi Matsui [1], Yoshihiro Sakoda, and Kyoko Tsukiyama-Kohara[1, 2*]

Supplementary Table 1. Result of microarray analysis. Cy3-labeled B10 RNA and cy5-labeled PYC-treated B10 RNA were characterized and spotted based on the gProcessed and rProcessed signals. Changes in gene expression of two-fold or more are shown. Red and green highlights show genes upregulated and downregulated upon PYC treatment, respectively. [Provided as Excel documents and Supplementary Dataset file]

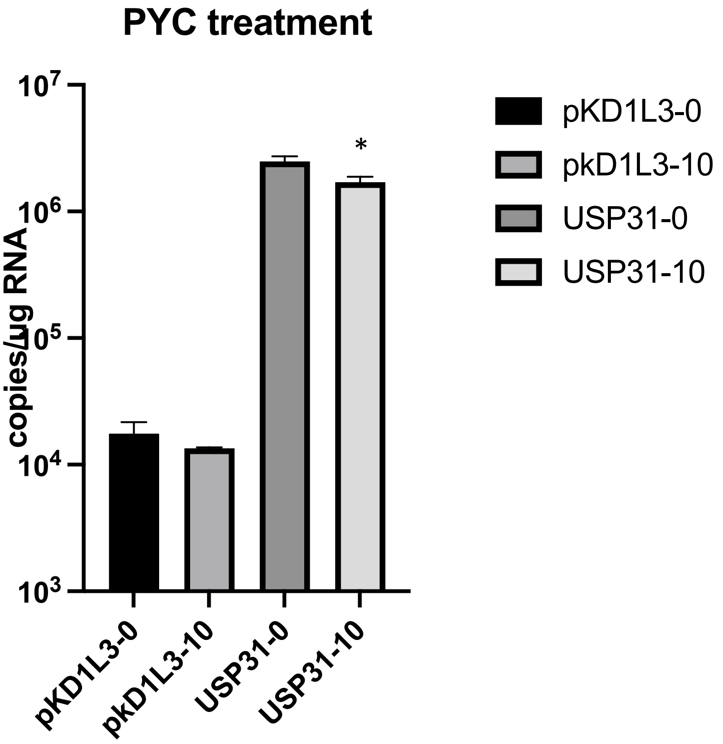

Supplementary Figure S1. Change in PKD1L3 and USP31 mRNA expression after PYC treatment. PKD1L3 and USP31 mRNA levels were quantified by qRT-PCR in cells with or without PYC treatment (10 µg/mL) for 72 h. *P = 0.034. Vertical bars indicate the SD.

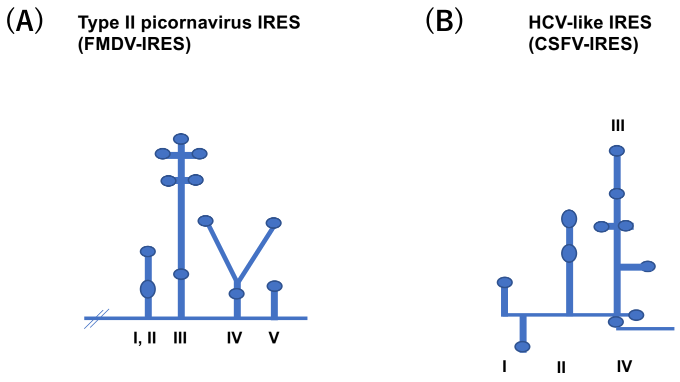

Supplementary Figure S2. Secondary structure of FMDV-IRES and CSFV-IRES. (A) Secondary structure of FMDV-IRES (type II picornavirus IRES). Domains I–V are shown. **(B)** Secondary structure of CSFV-IRES (HCV-like IRES) Domains I–IV are indicated.
